# Review of Random Phase Encoding in Volume Holographic Storage

**DOI:** 10.3390/ma5091635

**Published:** 2012-09-17

**Authors:** Wei-Chia Su, Ching-Cherng Sun

**Affiliations:** 1 Graduate Institute of Phonics, National Changhua University of Education, Changhua 500, Taiwan; E-Mail: wcsu@cc.ncue.edu.tw; 2 Department of Optics and Photonics, National Central University, Chung-Li 320, Taiwan

**Keywords:** volume holographic storage, random phase encoding, optical security, optical encryption, optical sensing, optical interconnection, spatial filtering

## Abstract

Random phase encoding is a unique technique for volume hologram which can be applied to various applications such as holographic multiplexing storage, image encryption, and optical sensing. In this review article, we first review and discuss diffraction selectivity of random phase encoding in volume holograms, which is the most important parameter related to multiplexing capacity of volume holographic storage. We then review an image encryption system based on random phase encoding. The alignment of phase key for decryption of the encoded image stored in holographic memory is analyzed and discussed. In the latter part of the review, an all-optical sensing system implemented by random phase encoding and holographic interconnection is presented.

## 1. Introduction

Volume holographic storage has received increasing attention in the field of optical memory and information processing owing to its high access rate, high selectivity and large storage capacity [1,2,3]. The ability of parallel processing in volume holographic storage is an excellent characteristic that the current commercial storage technology cannot compete with. To achieve the theoretically maximum storage density in volume holograms, various storage configurations and multiplexing techniques are proposed to store multiple images at the position in a medium [4,5,6,7,8,9,10,11,12,13,14,15,16,17,18,19]. Among these techniques, random phase encoding is notably worthy since it provides an additional function for data security [11,12,13,14,15,16,17,18,19,20,21,22,23,24,25,26,27]. In general, random phase encoding is performed by a phase plate with surface variation, such as a ground glass or other type of diffuser. Using a ground glass may be the simplest way to perform random phase encoding. The unpredictable surface variation enables a ground glass to be a key in data encryption. The decryption of the data cannot work if the key is lost except when a duplicated phase key is obtainable [28]. However, several main characteristics associated with data security are difficult to duplicate in the phase plate and alignment in the readout process [29].

Early study of random phase encoding with holographic storage can be referred to the work made by LaMacchia and White [11] in 1968.This technique is helpful to increase multiplexing selectivity for thinner holographic materials which lead to worse Bragg selectivity. Related research presented by Bashaw, Heanue, Aharoni, Walkup, and Hesselink [30] in 1994 has shown that random phase multiplexing has worse performance on cross talk noise than other multiplexing techniques. For thicker recording material, the cross talk noise of random-phase-multiplexed holograms can be suppressed owing to the Bragg effect. However, the suppression effect depends on the material’s thickness. In spite of worse cross talk performance, it is still possible to take the benefits of random phase multiplexing to obtain an improved density because storage capacity for holographic memory is not limited simply by cross talk noise. Dynamic range of recording material, multiplexing selectivity and scattering noises of reconstruction hologram are the other important factors and should be considered simultaneously. Accordingly, a storage density of 4.6 Gigapixels/cm^3^ based on random phase multiplexed holographic memory has been reported by He *et al.* [12]. An effective analysis model for estimating diffraction selectivity of random phase multiplexing was first proposed by Sun *et al.* [14] in 2000.

**Figure 1:**
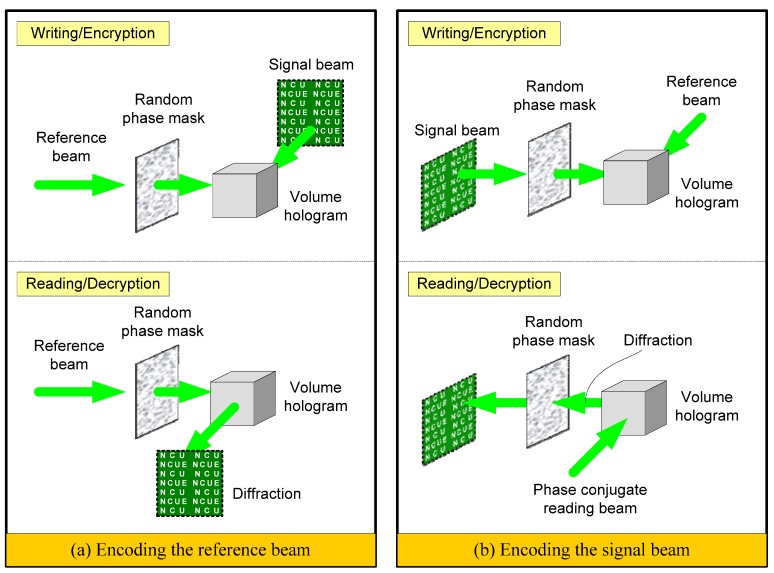
Two different schemes of encrypted holographic storage using random phase encoding.

Security data storage with random phase encoding accompanied by orthogonal-phase multiplexing was presented by Heanue, Bashaw, and Hesselink [16] in 1995 and by Denz, Muller, Visinka, and Tschudi [17] in 1999. Security holographic data storage implemented by random phase encoding becomes one attractive important issue due to the growing demand for security protection of information. In general, security holographic data storage implemented by random phase encoding can be accomplished in two approaches, as shown in Figure 1. In the first approach, the reference beam is propagated through a random phase generator, resulting in random phase distribution of the transmission wave [11,12,13,14,15,16,17,18,20]. Holographic multiplexing storage can be performed by shifting the phase mask to generate a series of uncorrelated reference beams [11,12,13,14,15] or by using orthogonal-phase multiplexing [16,17]. With holographic techniques, data is multiplexed in the same volume of recording medium. Each hologram corresponds to a specific reference wavefront. To retrieve the data, the user must use the same random-phase plate located at the correct position of reference arm to access the holographic memory.

In the second approach to security holographic data storage, based on random phase encoding, the object beam is encrypted by a random phase plate [19,31,32,33,34,35,36,37,38]. The characteristic of the second approach is that the original image can be converted into a random-noise-like image directly. Holographic multiplexing storage in this way is performed by recording the interference of encoded object beams and a series of reference beams with angular multiplexing [36,37,38]. The user can read out the encoded signal stored in the volume hologram by using a phase conjugate wave of the reference. Thus, a conjugate wave of the stored signal is diffracted in a reverse way to the original signal beam. The stored information carried by the readout signal beam can be retrieved only if the same random-phase plate is put at the same location of the signal arm for decryption. In addition, a good-fidelity phase-conjugated reading light is required for obtaining a high-quality decrypted image in a practical optical system [19]. However, in optical implementation, the random phase encoding system requires extremely precise alignment in the decryption process. The encrypted image cannot be decrypted if the decrypting phase mask deviates a certain distance from the matching position. Accordingly, Matoba and Javidi proposed an advanced encrypted concept [34]. In this system, not only the phase information but also the positions of the phase masks are used as encryption keys. Therefore, the shifting selectivity of phase masks offers important information for alignment and repositioning of the decryption phase key in a practical system. The theoretical analyses on lateral shifting selectivity were first discussed by Unnikrishnan, Joseph, and Singh [33] in 1998, and by Matoba and Javidi [34] in 1999. Later, the affect of the finite size of recording medium on the lateral sifting tolerance and theoretical analysis on longitudinal shifting tolerance was presented by Su and Lin in 2004 [37].

To study random phase encoding in holographic storage, one of the key characteristics is the Bragg selectivity or diffraction selectivity. In random phase encoding for holographic storage, high shifting selectivity can be performed in three dimensions [13,14,19,20,29,33,34,37]. Accordingly, encoding on reference or signal as described above provides a promising security for holographic storage. Unfortunately, high selectivity also leads to a tough task for optical alignment in the decoding process. Once the location of the authorized key in the reading process deviates from that in the writing process, it is a challenge to relocate the key to the location for decryption. Therefore, the alignment of the random phase plate becomes an important issue when optical random phase encoding is applied to a volume hologram. To solve this problem, a correlator-aided system for alignment of the random phase plate has been proposed and demonstrated [29]. Another practical issue for volume holograms with random phase encoding is that a crisis may occur when the phase key is damaged or lost. Duplication of the phase key is an essential demand for practical applications. A useful and effective way to reproduce such a phase key has also been proposed and demonstrated [28].

Another characteristic of high diffraction selectivity in holographic storage with random phase encoding is the application of optical sensing and optical interconnection [39,40,41,42,43,44]. The basic principle of optical sensing and optical interconnection based on volume holographic storage is first constructing a holographic memory, and then using the constructed holographic memory to serve as filters. The holographic memory is regarded as a database which records the interconnections between the incident waves and its corresponding output diffractions. In the operation process, the holographic database plays a role of filter to compare the access waves with the stored reference waves. If the access wave matches one of recorded reference waves in the database, a corresponding diffraction will be obtained through interconnection.

A holographic database for holographic interconnections or sensing can be done, accompanied by use of a multiplexing technique. And diffraction selectivity in these systems is the most important parameter which affects the multiplexing capacity of a holographic database and affects the sensor precision of a sensing system. We can find an early study of holographic interconnections focusing on the angular multiplexing technique [45,46]. However, in such schemes, the angular selectivities in the horizontal and vertical directions are quite different. To overcome the problems, Lee and Sang [40,47] proposed a new scheme of optical interconnection with random phase encoding. Recently, a new approach to perform a 2D-2D image interconnection with use of random phase encoding is proposed by Sun and his group [39,41].

Accordingly, random phase encoding has been applied to optical information processing, including holographic multiplexing [11,12,13,14,15,18,20], security data storage [11,12,13,14,15,16,17,18,19,20,21,22,31,32,33,34,35,36,37,38], optical sensing and optical interconnection [39,40,41,42,43,44]. In this paper, we review the studies related to random phase encoding in volume holography. In Section 2, we will first discuss how the system parameters affect the multiplexing selectivity of a volume hologram with random phase encoding. In Section 3, image encryption issues based on random phase encoding for security holographic storage are discussed. In Section 4, we review and discuss applications of fiber sensing in volume hologram with random phase encoding and holographic interconnections. In section 5, concluding remarks are made.

## 2. Random Phase Multiplexing

Random phase multiplexing was shown useful in increasing the holographic storage capacity in a thin holographic material, where the Bragg selectivity is not applicable [11]. In a practical holographic storage system, a general approach is to multiplex holograms in a volume hologram for higher storage capacity. However, the achievable storage density is limited by several other parameters, including the dynamic range of the material, signal-to-noise ratio of the diffracted signal, and the multiplexing capacity allowed by the multiplexing technique used. Among these factors, multiplexing is a key factor in determining the readout algorithm. One method to enhance multiplexing capacity is to use random-phase-encoded reference waves to obtain higher multiplexing selectivity. It has been shown that using random-phase-encoded reference waves in volume holographic storage can enhance the shifting, angular and wavelength selectivity [13,14,38,48,49,50,51,52,53,54,55,56]. Random phase encoding of reference waves by using ground glass for holographic multiplexing storage can be implemented by shifting or rotating the ground glass itself. The selectivity is analyzed in the following.

### 2.1. Shifting Selectivity

**Figure 2:**
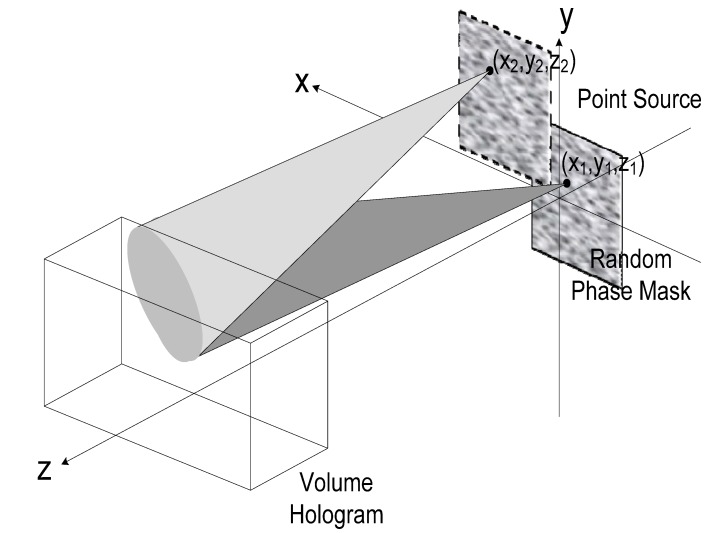
Schematic diagram of the shifting random phase encoding for volume hologram.

When a plane wave passes through a ground glass, the wavefront behind the glass can be treated as a superposition of the wavefronts emerged from a set of point sources with random-distributed initial phases as shown in Figure 2. Thus, we can write the composite wavefront on the hologram plane

(1) W(x3,y3)=∫−d2d2∫−d2d2A⋅exp{jϕ(x1,y1)}⋅exp{jkr1}⋅dx1⋅dy1

where *d* is the size of the illumination region of the ground glass; *A* is the amplitude of each spherical wave and $r_{1}={\left\{{\left(x_{3}-x_{1}\right)}^{2}+{\left(y_{3}-y_{1}\right)}^{2}+{\left(z_{3}-z_{1}\right)}^{2}\right\}}^{1/2}$ is the distance between the ground glass and the hologram; $\phi \left(x_{1},y_{1}\right)$ is the initial phase of each point source, which is associated with the surface roughness of the ground glass, so $\exp\left\{j\phi \left(x_{1},y_{1}\right)\right\}$ is a random distributed function across the encoded wave. We assume that the hologram records the interference fringes written by a plane wave and the reference wave described in Equation (1). Now, another wavefront is applied to read the hologram. Based on VOHIL (volume hologram being integrator of light emitted from elementary light sources) model [57], which is an effective and useful algorithm for analyzing diffraction selectivity of volume holograms, we can express the diffraction as

(2) D=∫−ℓ2ℓ2∫−d2d2∫−d2d2∫−d2d2∫−d2d2|A|2⋅B⋅exp{jϕ(x2,y2)−jϕ(x1,y1)}×exp{jk(r2−r1)}⋅dx1⋅dy1⋅dx2⋅dy2⋅dx3

where ℓ is the thickness of the hologram; *A* and *B* are the amplitudes of the reading and the signal waves, respectively; *r*_2_ is the distance between the decoding ground glass and the hologram, which can be expressed as $r_{2}={\left\{{\left(x_{3}-x_{2}\right)}^{2}+{\left(y_{3}-y_{2}\right)}^{2}+{\left(z_{3}-z_{2}\right)}^{2}\right\}}^{1/2}$; $\phi \left(x_{2},y_{2}\right)$ is the initial phase of each point source on the ground glass used for encoding the reading wave. Once the ground glass encoding the reading wave is not the same as that encoding the reference wave, the diffraction is suppressed, due to destructive interference. Therefore, no obvious diffraction light can be observed. Therefore, in the following analyses, the ground glass used for encoding the reference is the same as that used for the reading waves, but the position could be different. The effective diffraction is caused only by the self-reading of each point source. Then the conditions of $\phi \left(x_{2},y_{2}\right)-\phi \left(x_{1},y_{1}\right)=0$ must be satisfied in Equation (2). Supposed the ground glass is shifted at a distance of $\Delta =\sqrt{\Delta x^{2}+\Delta y^{2}+\Delta z^{2}}$, where Δ*x*, Δ*y* and Δ*z* are the shifting in horizontal, vertical, and longitudinal directions, respectively. In the following, we discuss the cases when the ground glass is shifted in the lateral and longitudinal directions respectively.

#### 2.1.1. Lateral Shifting Selectivity

In this case, $\Delta x=x_{2}-x_{1}$, $\Delta y=y_{2}-y_{1}$, and Δz=0. Under the paraxial condition, $r_{1}\approx z_{0}$; $r_{2}\approx z_{0}$, where *z*_0_ is the distance between the ground glass and the crystal. Equation (2) can be rewritten

(3) D=∫−ℓ2ℓ2∫−d2d2∫−d2d2|A|2Bℓd⋅exp{jkz02z0[(x3−x1−Δx)2+(y3−y1−Δy)2−(x3−x1)2+(y3−y1)2]}⋅dx1⋅dy1⋅dx3

The solution of Equation (3) for the diffraction with respective to Δ*x* and Δ*y* can be obtained

(4) $$D={\left|A\right|}^{2}B\ell d\cdot \exp\left\{\frac{jk\left(\Delta x^{2}+\Delta y^{2}\right)}{2z_{0}}\right\}\cdot \exp\left\{\frac{-jk\left(\Delta y\cdot y_{3}\right)}{z_{0}}\right\}\cdot \text{sinc}\left(\frac{\Delta x\cdot d}{\lambda \cdot z_{0}}\right)\cdot \text{sinc}\left(\frac{\Delta x\cdot \ell }{\lambda \cdot z_{0}}\right)\cdot \text{sinc}\left(\frac{\Delta y\cdot d}{\lambda \cdot z_{0}}\right)$$

The diffraction intensity can be expressed as

(5) $$I\propto {\left|D\right|}^{2}={\left({\left|A\right|}^{2}B\ell d/z_{0}{}^{2}\right)}^{2}{\text{sinc}}^{2}\left(\frac{\Delta x\cdot d}{\lambda z_{0}}\right){\text{sinc}}^{2}\left(\frac{\Delta x\cdot \ell }{\lambda z_{0}}\right){\text{sinc}}^{2}\left(\frac{\Delta y\cdot d}{\lambda z_{0}}\right)$$

If the paraxial condition is satisfied, Equation (5) can be further simplified

(6) $$I\propto {\text{sinc}}^{2}\left(\frac{2\Delta x\cdot NA_{GG}}{\lambda }\right){\text{sinc}}^{2}\left(\frac{2\Delta x\cdot NA_{H}}{\lambda }\right){\text{sinc}}^{2}\left(\frac{2\Delta y\cdot NA_{GG}}{\lambda }\right)$$

Where

(7) $$NA_{GG}=\frac{d}{2z_{0}^{}}$$

(8) $$NA_{H}=\frac{\ell }{2z_{0}^{}}$$

Equations (5–8) are general solutions under weak coupling, and we can accordingly investigate its lateral shifting selectivity in the horizontal and vertical directions.

#### 2.1.2. Longitudinal Shifting Selectivity

In the case of Δx=0, Δy=0, *and* $\Delta z=z_{2}-z_{1}$, Equation (2) become

(9) D=|A|2⋅B⋅∑x3=−ℓ2ℓ2∑x1=−d2d2∑y1=−d2d2[z02+(y3−y1)2+(x3−x1)2]−12⋅[(z0+Δz)2+(y3−y1)2+(x3−x1)2]−12×exp{jk[(z0+Δz)2+(y3−y1)2+(x3−x1)2]12}⋅exp{−jk[z02+(y3−y1)2+(x3−x1)2]12}

**Figure 3:**
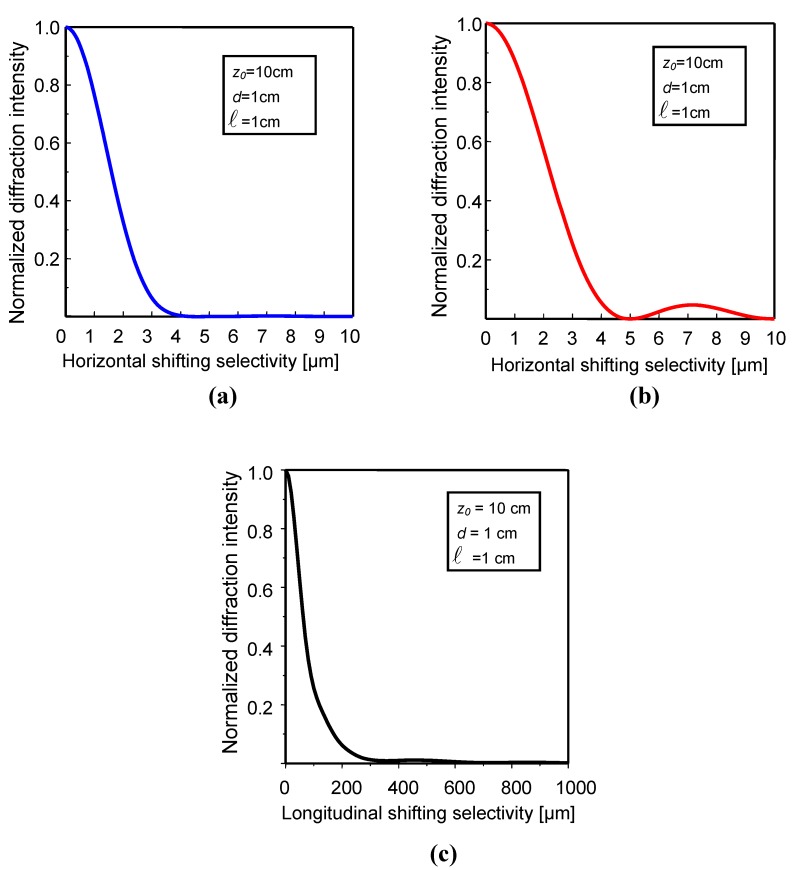
Theoretical shifting selectivity for random phase multiplexing in volume holographic storage.

The theoretical calculations of the shifting tolerance in horizontal, vertical, and longitudinal direction are shown in Figure 3.

The parameters used in the calculations are z_0_ = 1 cm, ℓ=1 cm, and λ=514.5 nm. The evaluation of the 3-D shifting tolerance is useful to random phase encoding for multi-layer storage. Assuming that the full shifting tolerance in three dimensions are $\Delta x_{s}$, $\Delta y_{s}$, and $\Delta z_{s}$, respectively, and the ground glass can be shifted in a range of $L_{x}\times L_{y}\times L_{z}$, then the multiplexing capacity is LxΔxs×LyΔys×LzΔzs. Based on VOHIL model, we have derived a general expression for the diffraction selectivity of the ground glass for random phase encoding. The mechanisms of 3-D selectivity are described theoretically, and the experimental results support those theoretical predictions [13,14]. From our analysis and corresponding experimental observation, a convenient method for increasing the shifting selectivity as well as the multiplexing capacity is to enlarge the illumination dimension on the ground glass and to shorten the distance between the ground glass and the volume hologram.

### 2.2. Rotational Selectivity

An alternative scheme, rotating a ground glass for holographic multiplexing, has been proposed [58,59]. The rotation selectivity is important, not only to holographic storage, but also optical sensing when the hologram is applied to spatial filtering [60,61,62]. Rotation selectivity of a volume hologram with random phase encoding can also be theoretically estimated, based on the VOHIL model.

**Figure 4:**
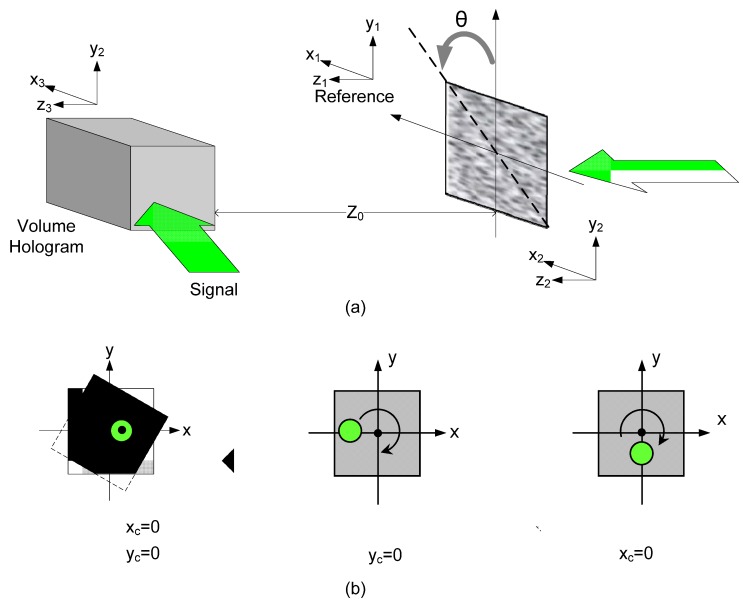
(**a**) Schematic diagram of the rotational random phase encoding for volume holograms; (**b**) Three different locations of the illumination area on the ground glass.

The schematic diagram of the rotation multiplexing is shown in Figure 4a. Since the wavefront behind the glass can be treated as a superposition of the wavefronts emerging from a set of point sources with random-distributed initial phases, we may express the composite wavefront on the hologram plane as Equation (1). In the following analysis, the ground glass used for encoding the reference is the same as that used for the reading waves, but the position is different owing to rotation of the ground glass.

Regarding rotational structure, the distance between the rotational center and the illuminated ground glass is one of the key factors. The holographic selectivity will be a function of the location of the laser spot on the ground glass. Figure 4b shows the three typical conditions of the illumination condition. If we denote the coordinates of $\left(x_{c},y_{c}\right)$ as the center of the illumination spot, and (0, 0) as the rotational center, the coordinates of each point of the ground glass after rotation can be expressed [54]

(10) $$\begin{array}{l} x_{2}=x_{1}\cdot \cos\left(\Delta \theta \right)-\left(y_{1}+y_{c}\right)\cdot \sin\left(\Delta \theta \right)-x_{c}\\ y_{2}=x_{1}\cdot \sin\left(\Delta \theta \right)-\left(y_{1}+y_{c}\right)\cdot \cos\left(\Delta \theta \right)-y_{c} \end{array}$$

**Figure 5:**
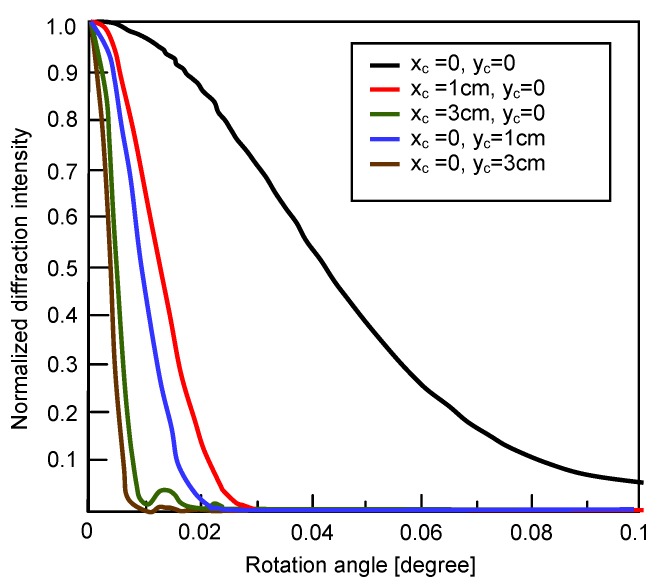
Rotational sensitivity of the volume hologram with random phase encoding.

In the reconstruction of the hologram, the same ground glass is used, but it is rotated with an angle Δθ, so the initial phase of the reference and the reading light is the same. Through the calculation of Equations (2) and (3), we can obtain the relative diffraction selectivity with respect to the rotation angle of the ground glass. Figure 5 shows the simulation result for different illumination diameters, where the wavelength is 514.5 nm, the distance ($z_{0}$) between the ground glass and the crystal and the hologram is 10 cm and the hologram dimension along the signal direction is 10 mm. We may find that the hologram is more sensitive to the rotation when the distance $z_{0}$ becomes smaller or the diameter of the illumination spot on the ground glass becomes larger. Besides, the location of the illumination spot is important to the Bragg selectivity. When the illumination spot is located at the y-axis other than (0, 0), the hologram will be most sensitive to the rotation of the ground glass.

## 3. Image Encryption for Volume Holographic Storage

Several image encryption algorithms have been proposed by use of optical random phase encoding, such as double random phase encoding [31,32,33,34,35,36,37,38], image encoding [63], and Fourier plane encoding [64]. Among these schemes, the technique of double random phase encoding uses two independent random phase masks located at the input and the Fourier planes to convert an original image into a random-noise-like pattern [31,32,33]. The random-noise-like pattern is so-called the encrypted image. Then, holographic storage of multiple encrypted images is implemented sequentially by recording interference of each encrypted image and its corresponding reference wave in a holographic recording medium [36,37,38]. This encryption technique shows good tolerance to data loss of the encrypted image and different types of noise [65,66,67,68]. In optical implementation, a phase conjugation readout algorithm must be used for image decryption in this scheme. In addition, the double random phase encoding system requires extremely precise alignment in the decryption process; otherwise, the encrypted image cannot be decrypted. Accordingly, an advanced encrypted optical memory system is proposed by shifting the random phase codes away from the input and the Fourier planes [34,35]. In the system, not only the phase information but also the positions of the phase masks are used as encryption keys. Therefore, the shifting selectivity of phase masks offers important information for alignment and repositioning of the decryption phase key in a practical system.

Our study shows that the lateral shifting selectivity of the decryption phase mask in the system depends on not only its correlation length, but also the dimension of recording medium and distance between hologram and phase mask. When the signal passes through the encrypted random phase mask, the wavefront behind the mask can be treated as a superposition of spherical waves emerging from the phase pixels on the mask. In the phase-conjugate reconstruction, the holographically recorded spherical wave diverging from each signal phase pixel fails to perfectly focus back onto itself and causes a blur because only a limited numerical aperture (NA) of the spherical wave was captured by the hologram. The numerical aperture comes from the hologram transverse size and its distance from the mask. We define $NA=l/2z_{0}$, where *l* is the transverse size of the crystal and $z_{0}$ is the distance between the phase mask and the hologram. We find the blur size is inversely proportional to the numerical aperture. When the numerical aperture becomes smaller, the blur size will become larger. If a mask with high spatial frequency is used in the system, the diffraction selectivity could depend on the blur size only. Therefore, when a mask with a high spatial frequency is used in the system, we can increase the distance between the crystal and the phase mask or reduce the hologram size to obtain a larger blur size and then obtain a larger shifting tolerance.

**Figure 6:**
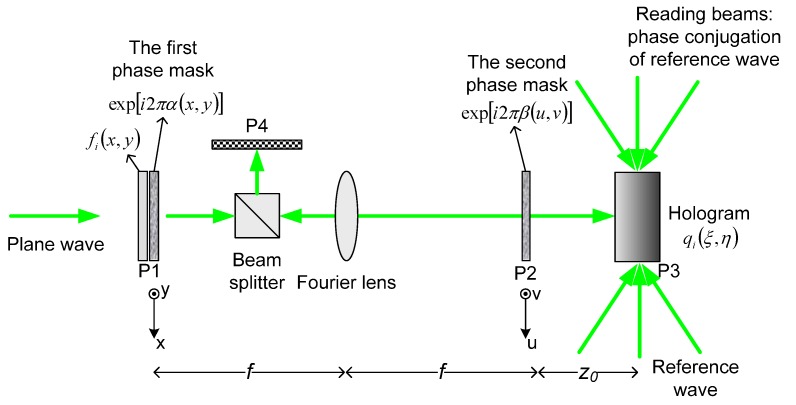
Holographic storage of encrypted image that uses double-random phase encoding.

Figure 6 shows the schematic diagram of the image encryption, based on a double random phase encoding technique for holographic storage. Let $f_{i}\left(x,y\right)$ denote the *i*th image to be encrypted and $q_{i}\left(\xi ,\eta \right)$ denotes the *i*th encrypted image. α(x,y) and β(u,v) represent two independent random functions, which are uniformly distributed in [0,1]. Here (x,y), (u,v), and (ξ,η) denote the spatial domain coordinates in input plane P1, Fourier plane P2, and recording plane P3, respectively. The double random phase encryption of the image $f_{i}\left(x,y\right)$ is obtained by the following operations. First, the image $f_{i}\left(x,y\right)$ contacted with a random phase mask exp[i2πα(x,y)] is placed on the input plane P1 and is illuminated by a coherent plane wave. Second, on the Fourier plane P2, the Fourier transform of the product $f_{i}\left(x,y\right)\exp\left[i2\pi \alpha \left(x,y\right)\right]$ is multiplied by the second random phase code exp[i2πβ(u,v)]. Finally, through Fresnel diffraction approximation, the encrypted image $q_{i}\left(\xi ,\eta \right)$ is obtained on the hologram plane P3. Such encrypted data $q_{i}\left(\xi ,\eta \right)$ is stored in a volume holographic medium, such as a LiNbO_3_ photorefractive crystal, with a reference plane wave. To store more frames of data, angular multiplexing can be employed. In the decryption process, the phase conjugate of the reference beam is used to read the stored encrypted data in the crystal. The data of *i*th stored image can be reconstructed when the readout beam is incident at a correct angle. The conjugate diffracted light will go back to the Fourier plane. If the decryption phase mask is the same as the original one, we can obtain a well-decrypted pattern. If a different decryption phase mask were to be used in the decryption process, the output image in the plane P4 would remain as white noise.

**Figure 7:**
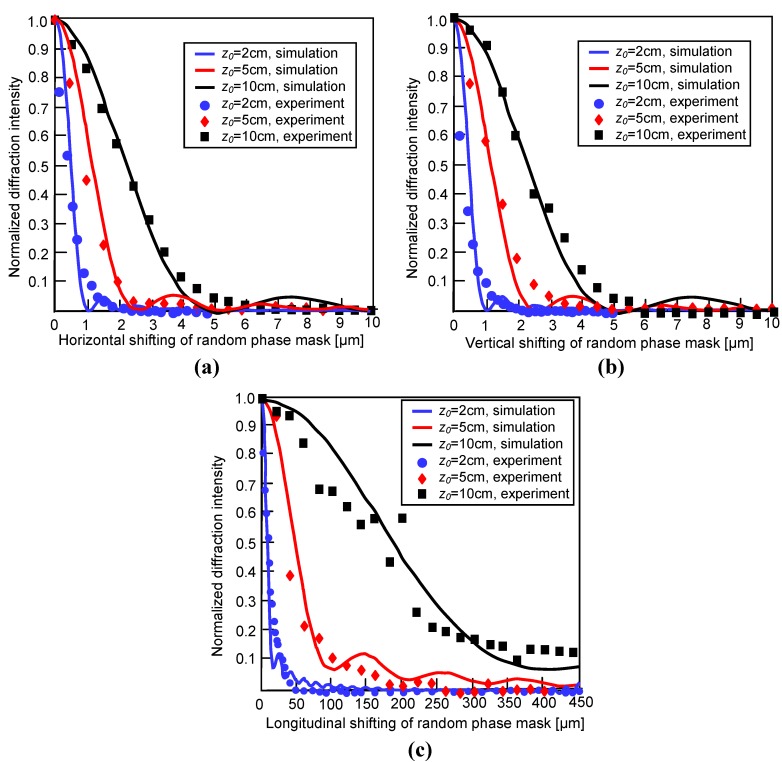
Diffraction selectivity of the ground glass for holographic storage of encrypted images.

If the decryption phase mask only shifts in the horizontal direction or the vertical direction, the decryption shifting selectivity becomes a cross correlation between the original wavefront and the broadened wavefront. Then the diffraction selectivity can be determined

(11) $$\eta (\Delta x,\Delta y)={\left\{\exp\left[i2\pi \beta \left(\Delta x,\Delta y\right)\right]⊕\left[\exp\left[i2\pi \beta \left(\Delta x,\Delta y\right)\right]⊗\text{sinc}(\frac{l_{x}\Delta x}{\lambda z_{0}})\text{sinc}(\frac{l_{y}\Delta y}{\lambda z_{0}})\right]\right\}}^{2}$$

where ⊗ is the convolution operation and ⊕ is the correlation operation. The sinc function represents the point-spread function of each point source on the phase mask. The practical lateral diffraction selectivity becomes a convolution between the correlation function and the point-spread function. The theoretical calculation of the shifting tolerance in horizontal direction and vertical direction based on Equation (11) are shown in Figure 7a and Figure 7b. The parameters in the calculations are $l_{x}=1\;cm$, $l_{y}=1\;cm$, and λ=514.5 nm when $z_{0}=2\;cm$, 5 cm, and 10 cm, respectively. The correlation length of the ground glass used in the simulation is 1 μm. In addition, the diffraction selectivity strongly depends on the distance between hologram and phase mask. When the distance is increased, the larger shifting tolerance can be obtained.

The same condition also describes the longitudinal point-spread function of each decrypted point source on the phase mask. Therefore, the longitudinal diffraction selectivity is obtained from the longitudinal point-spread function. When $l_{x}=1\;cm$, $l_{y}=1\;cm$, and λ=514.5 nm, the theoretical calculation of diffraction selectivity in the longitudinal direction for *z*_0_ = 2 *cm*, 5 *cm*, and 10 *cm* is shown in Figure 7c. We find that the shifting tolerance is proportional to the distance between hologram and the phase mask. From theoretical analysis and experimentally investigated results, we can conclude that the lateral diffraction selectivity is determined by the convolution of the point-spread function induced by the crystal and the correlation function of the mask. The longitudinal diffraction selectivity depends on the longitudinal point-spread function. Therefore, enlarging the point-spread function is helpful to increase the shifting tolerance. From the analyses, there are two kinds of methods to enlarge the point-spread function. One is to increase the distance between the crystal and the phase mask and the other is to reduce the crystal size. The diffraction selectivity derived here offers important information for alignment and repositioning of the decrypted phase key in practical optical implementations.

## 4. All Optical Fiber Sensors

**Figure 8:**
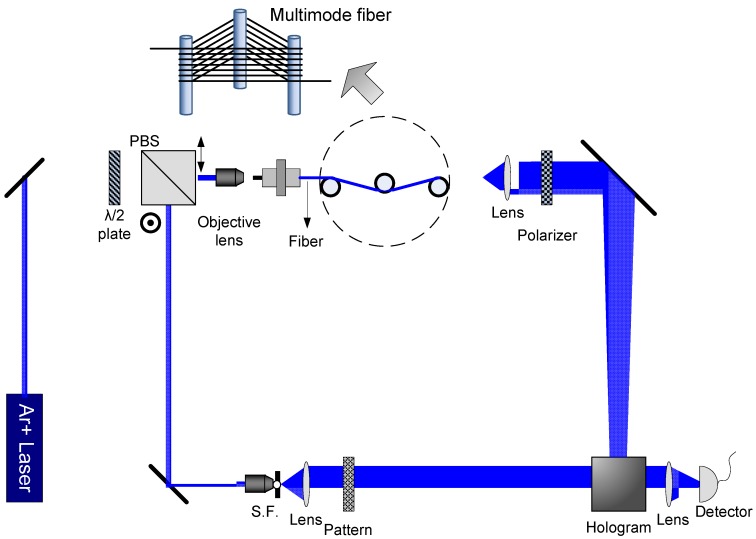
Schematic diagram of a fiber sensing system.

**Figure 9:**
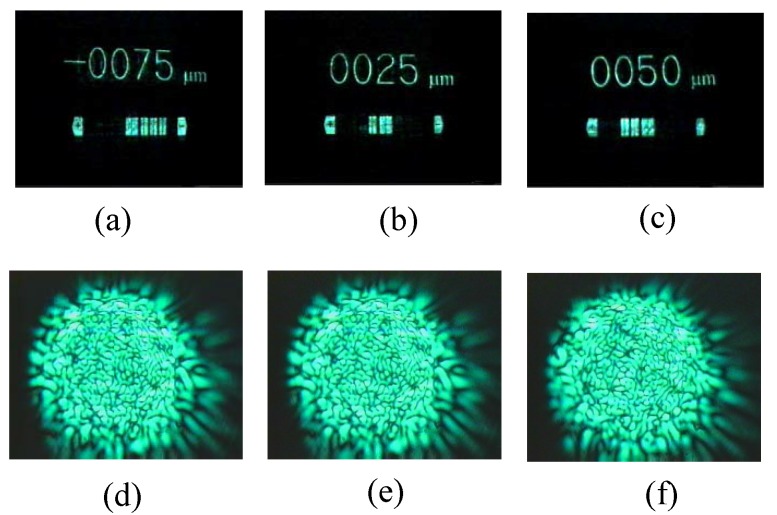
(**a**–**c**) are three selected diffraction images from the sensor system; (**d**–**f**) are the reference speckles corresponding (**a**–**c**), respectively.

Random phase can be easily generated through a ground glass or a multi-mode fiber, which can be applied to precisely sensing. The operation mechanism of an optical sensing system, based on random phase encoding, relies on the holographic interconnections [35,36,37,38,39,40,41,42,43,44]. For a multi-mode fiber, any environmental perturbation could cause an optical path length change of each propagating mode, and consequently the phase and intensity distribution of speckle is changed. Based on the variation of speckle, several optical fiber testing algorithms have been proposed [39,69,70,71]. Figure 8 demonstrates an all-optical fiber sensing system where a multi-mode fiber is the sensing element, and it provides various speckle patterns that are applied to random phase encoding and optical interconnection. A database in volume holograms should be constructed to boot the system. In the construction process, a specific speckle corresponding to a specific perturbation on a multiple-mode fiber is used as the reference of a volume hologram, and a pattern indicating the amount of the perturbation is used as the signal. We can perform holographic storage with random phase multiplexing once the incoming speckle becomes uncorrelated with the previous one. The stored holograms in the crystal could be regarded as a database used for the interconnections between the incoming speckle and the corresponding output pattern. In the sensing process, once a specific speckle from the sensing fiber is incident on the crystal, the volume hologram can automatically compare the phase of the incoming speckle with the stored ones. If the incoming speckle matches one in the database, a pattern will be diffracted through the interconnection. When we stirred the fiber with our fingers, we observed a series of output patterns indicating the amount of the perturbation in real-time. The diffraction signal has linearity and repeatability with the perturbation. The speed for the signal transmission, data processing and the display of the result is as fast as light. Some examples of diffraction images and the corresponding speckles in the system are shown in Figure 9. In this way, we can not only demonstrate the principle of this fiber sensing, but also realize an all-optical fiber sensing system, where optical storage, optical sensing, optical interconnection, optical computing and display are all undertaken by optical means.

## 5. Conclusions

In this review paper, we have first presented the three-dimensional shifting selectivity of volume holograms based on random phase encoding with ground glass. The diffraction characteristic is caused by the phase difference between the reference and reading lights and can be analyzed by using the VOHIL model. We find that the shifting selectivity is different for different shifting directions, which include laterally horizontal, laterally vertical, and longitudinal directions. The shifting selectivity depends on the diameter of the illumination region on the random phase plate, the thickness of the hologram and the distance between them. We have then also shown rotational selectivity of volume holograms based on random phase encoding with a ground glass. By controlling the parameters including rotational center, effective numerical aperture of both volume hologram and the ground glass, we can obtain different rotational selectivity applied to random phase encoding in volume holographic storage. Selectivity of random phase encoding offers important information to estimate the multiplexing capacity of volume holographic storage. Accordingly, the theoretical calculation on multiplexing selectivity developed, based on the VOHIL model, is very helpful in practical holographic storage systems. In addition, the ground glass used for generating random phase also offers a security function. To retrieve the data, the user must have the same phase key and put it on the same position as during writing. Accordingly, the ground glass served as the phase key for securing the stored data, and therefore volume holographic storage implemented by random phase multiplexing leads to an inherently secure memory.

In the second part of the review manuscript, we have discussed an alternative security approach for holographic storage. In this approach, image encryption is achieved by converting the original image into stationary white-noise data by random phase encoding. The storage of the encrypted image is then recorded holographically with other multiplexing techniques, such as angle multiplexing. The encryption and decryption process implemented by random phase encoding is reviewed and then the shifting tolerance of phase key during the decryption process is theoretically analyzed. The lateral shifting selectivity of the decryption phase mask in the system depends not only on its correlation length, but also the dimension of recording medium and the distance between phase mask and the hologram. The longitudinal diffraction selectivity is inversely proportional to the dimension of recording medium and proportional to the distance between hologram and the phase mask. The diffraction selectivity derived in this paper offers important information for alignment and repositioning of the decryption phase key in practical optical implementations.

In the last part of the review, we have demonstrated an all-optical fiber sensing system based on random phase encoding and volume holographic interconnection. In this system, data storage, signal processing and display are all achieved by optical means. Without the optical parallelism performed by random phase encoding in a volume hologram, such a demonstration seems impossible. We find random phase encoding is not only useful in holographic storage but also in optical encryption and optical sensing. We hope this review article will stimulate further research in volume holograms with random phase encoding.
